# Is inflammatory chronic obstructive pulmonary disease a coronary heart disease risk equivalent? A longitudinal analysis of the third National Health and Nutrition Examination Survey (NHANES III), 1988–1994

**DOI:** 10.1186/1471-2466-14-195

**Published:** 2014-12-05

**Authors:** Donna R Parker, Jonathan Liu, Mary B Roberts, Charles B Eaton

**Affiliations:** Brown Center for Primary Care and Prevention, Memorial Hospital of Rhode Island, Pawtucket, USA; Department of Family Medicine, Alpert Medical School of Brown University, Providence, USA; Department of Epidemiology, Brown University School of Public Health, Providence, USA; Alpert Medical School of Brown University, Providence, USA

## Abstract

**Background:**

Evidence suggests that there is an association between chronic obstructive pulmonary disease (COPD) and coronary heart disease (CHD). An important etiological link between COPD and CHD may be an underlying systemic inflammatory process. Given that COPD patients are at greater risk of cardiovascular mortality, understanding the burden of CHD on COPD patients could permit future risk attenuation.

**Methods:**

Longitudinal cohort analyses of the Third National Health and Nutrition Examination Survey from 1988–1994 were performed. 3,681 individuals ≥40 years of age with good quality spirometry data were included. Participants were divided into 5 groups: 1) no COPD, no CHD; 2) COPD without inflammation, no CHD; 3) COPD with inflammation, no CHD; 4) CHD only, and 5) CHD + COPD. A novel “inflammatory” COPD designation included those with COPD and clinical evidence of inflammation (i.e., CRP ≥95.24 nmol/L).

**Results:**

The risk for CHD mortality was significant only for the CHD group (HR 5.56, 95% CI 3.24-9.55) and the COPD + CHD group (HR 5.02, 95% CI 2.83-8.90). Similarly, the risk for cardiovascular disease (CVD) mortality was significant only for the CHD group (HR 4.25, 95% CI 2.70-6.69) and the CHD + COPD group (HR 4.12, 95% CI 2.60-6.54) after adjusting for nonmodifiable CHD risk factors (age, gender, race/ethnicity, family history of CHD). After adjusting for modifiable CHD risk factors (diabetes, BMI, physical activity, systolic blood pressure, cholesterol, and smoking), hazard ratios of the two groups remained similar but attenuated. For total mortality, the risk was significant for the four groups: the non-inflammatory COPD group; the COPD with inflammation group, the CHD group, and the COPD + CHD group.

**Conclusions:**

Our study did not confirm that inflammatory COPD may be a CHD risk equivalent. However, due to the small size of the “inflammatory” COPD group, further prospective replication and validation is needed. Moreover, given that COPD results from inflammation, the systemic inflammation associated with COPD may have worsened comorbid conditions and may have lead to the increased total mortality found in the COPD with inflammation and COPD + CHD groups which requires further investigation.

## Background

Chronic obstructive pulmonary disease (COPD) is an increasingly prevalent disease affecting 11.8 million people in the United States with evidence that it is underdiagnosed and may actually affect as many as 24 million individuals [1, 2]. According to the Centers for Disease Control and Prevention (CDC), COPD recently overtook cerebrovascular diseases to become the third leading cause of death in the US, lagging only behind diseases of the heart and malignant neoplasms [3]. Of concern is that while COPD was originally predicted to reach third place by 2020, this was achieved in 2008 [4]. In fact, while the death rate from heart disease and stroke has dropped, COPD mortality rates doubled from 1970 to 2002 [5]. COPD not only contributes to morbidity and mortality but also represents a large economic burden to the US, with a 2010 estimated cost of $49.9 billion [6].

The causes of death in COPD remain poorly understood, as studies reveal conflicting data. Etiologies such as lung cancer [7], respiratory failure [8], and cardiovascular disease [9], have all been found to be the most common cause of death in COPD patients. Death from COPD itself was found to be the most common cause (59.8%) in one large population study from England with diseases of the circulatory system in second place [10]. Further, while death from respiratory disease and lung cancer are falling in COPD patients, death due to circulatory system causes is on the rise [11]. In fact, evidence suggests that patients with COPD may have a two to threefold increased risk for coronary heart disease death.

Research has revealed that COPD has been found to be associated with persistent low-grade systemic inflammation [12, 13]. Systemic inflammation is present not only in individuals with reduced lung function but is also present in various other chronic diseases including coronary heart disease (CHD) [14, 15]. Given the burden of lung and cardiovascular diseases (CVD) and emerging data suggesting a direct link between lung injury and inflammation and CHD, more research is needed to quantify the burden that CHD represents in individuals with COPD. For this study, we examined this association using data from the Third National Health and Nutrition Examination Survey (NHANES III), 1988–1994. We hypothesize that COPD is not only a risk factor for CHD but may, in fact, be a CHD risk equivalent.

## Methods

### Study population

NHANES III is a cross-sectional survey conducted between 1988 and 1994 by the National Center for Health Statistics (NCHS). NHANES III provides a nationally valid estimate of health and nutrition status among a representative group of civilian, noninstitutionalized US residents. Participants signed a consent form during the survey. NHANES Institutional Review Board (IRB) approval was obtained.

In order to provide accurate estimates, young children, the elderly, non-Hispanic blacks and Mexican Americans were oversampled. Sampling methods and design have been previously described [16, 17]. Each participant was assigned a weight to allow for calculation of population-level estimates. NHANES III includes household interviews, physical exams in the Mobile Examination Center (MEC) and home examinations when participants could not reach the MEC.

Since COPD is uncommon in younger age groups, we limited our study population to participants ≥40 years old with good quality, reliable spirometry data based on the criteria of the American Thoracic Society [18]. Exclusion criteria included individuals <40 years old, pregnant women, Asians (due to a limited number), home-assessed or unreliable spirometry, and a positive response to either of the following questions (“Have you had a cough, cold, or other acute illness in the past few days?” or “In the past three weeks have you had any respiratory infections, such as flu, pneumonia, bronchitis, or a severe cold?”). Our study population included 3,681 individuals who met these eligibility criteria.

### Mortality follow-up

We evaluated coronary heart disease as well as cardiovascular disease and all-cause mortality as outcomes. Mortality follow-up data for NHANES III participants was available through December 31, 2006. Mortality status was obtained through the National Death Index (NDI) via probabilistic record matching conducted by the NCHS. Matching criteria included social security number, first and last name, middle initial, birth date, gender, race, marital status, state of birth and state of residency [19]. Mortality due to CHD as the primary cause of death was classified by using the International Classification of Disease 10^th^ Revision (ICD-10) 120.0-125.9. Mortality due to CVD was classified by ICD-10 codes: 120.0-125.9, 160–178 (ischemic stroke). Number of months follow-up was determined from the “Person Months of Follow-up from Interview Date” variable in the mortality dataset, which was used in the time to death analysis [20].

### Measurements and variables

In depth documentation of NHANES III laboratory and sample collection methods have been previously described and are briefly outlined below [21].

Age, gender and race information were collected during the home interview. Race was self-classified as white, black, non-black Hispanic or other. The self-report of physical activity included a questionnaire that evaluated the frequency and intensity of leisure time physical activities including dancing, jogging, running, bicycling, swimming, aerobics, and other activities. Intensity of the physical activity was expressed as a weekly metabolic equivalent (MET). METs are defined by increasing energy expenditure with MET = 1 corresponding to the resting metabolic rate and MET = 10 representing running at 6 mph [22].

Insurance status was determined during the home interview and includes coverage under Medicare, Medicaid, CHAMPUS, CHAMPVA, VA and other private or employer/union health plans. Marital status was measured as the percent of participants who were partnered, including those who were married or living as married. Education was measured as the percent of participants who did not complete high school (<12 years of schooling). Income was determined as the percent of participants with a total combined family income over the last year amounting to less than $20,000. Employment status was based on responses to questions asking if the participant had a job or business or if they worked within the past 2 weeks.

Blood pressure was reported as the average of five different measurements, taken by mercury sphygmomanometers (W. A. Baum Co., Copiague, NY) using standard technique [23]. The designation of hypertension (HTN) was given to those who were taking anti-hypertensive medications or those with a positive response to the question “Were you told on 2 or more different visits that you had hypertension, also called high blood pressure?”

Diabetes status was defined by self-report or prior diabetic medication use. BMI was calculated as weight in kilograms divided by the square of height in meters with height rounded to the nearest 0.1 cm and weight to 0.01 kg.

Smoking status was divided into several categories including: current smokers, past smokers, never smokers and ever smokers. Current smokers were defined by answering “yes” to questions: “Do you smoke cigarettes now?” and “Have you smoked at least 100 cigarettes in your lifetime?” Past smokers included those who do not smoke cigarettes now but smoked over 100 cigarettes in their lifetime. Never smokers were those who smoked less than 100 cigarettes in their lifetime. Ever smokers were determined by combining past and current smokers. Number of cigarettes per day was also measured. A participant was considered exposed to environmental tobacco smoke (second-hand smoke) if he/she currently smoked or lived with someone who smoked in the home.

Alcohol consumption was assessed through questions from the household interview asking how many times over the past month participants drank beer, wine and hard liquor. Frequency across all three alcohol types was summated and expressed as number of drinks per week and dichotomized to “any alcohol” or none.

Clinical symptoms of cough and phlegm were determined by a positive response to questions which asked participants if they coughed or produced phlegm on most days for 3 consecutive months or more. Shortness of breath was assessed through interview as being “troubled by shortness of breath when hurrying on level ground or walking up a slight hill”.

Diagnosis of COPD was based on spirometry. Spirometry was performed in a designated room in the MEC devoted to spirometry using a customized Ohio Sensormed 827 dry rolling seal spirometer [24]. Calibration was performed at the start of each session and participants were coached during the procedure so as to produce the best results. Participants were encouraged to blow for at least six seconds as required by ATS guidelines, thus measuring forced vital capacity (FVC). The session was complete when five acceptable trials were obtained, with a maximum of eight [24]. The tests were reviewed by two senior technicians and classified for reliability. All unacceptable maneuvers were excluded before reproducibility calculations were performed [24].

We used an FEV1/FVC ratio (forced expiratory volume in one second/forced vital capacity) of <0.70 to define airflow obstruction. Mild, moderate, severe, and very severe airflow obstruction were defined as respectively: stage 1 -FEV1 ≥ 80% of predicted; Stage 2 -50% ≤ FEV1 < 80% predicted; Stage 3 - 30% ≤ FEV1 < 50% predicted; and Stage 4 -FEV1 < 30% predicted based on the GOLD COPD Guidelines [25] and the predicted FEV1 based on the equation developed by Hankinson et al. [26].

CHD diagnosis was based on an affirmative response to the interview question: “Has your doctor ever told you that you had a heart attack?” Participants were classified into CHD risk groups based on the number of CHD risk factors or risk equivalents they had [27].

Laboratory analyses: fasting blood samples were obtained in the MEC or during the home interview. All blood samples were frozen and shipped on dry ice to laboratories for analysis. Upon arrival at the laboratories, frozen specimens were initially stored at -20°C, refrigerated samples were stored at 4-8°C, and frozen specimens with delayed analysis were stored at -70°C or lower [21].

Triglycerides (TG), HDL cholesterol (HDL-c), and total cholesterol (TC) analyses were performed by the Hitachi 704 Analyzer (Boehringer Mannheim Diagnostics, Indianapolis, IN). LDL cholesterol (LDL-c) was not calculated from these measurements because a certain percentage of the population did not fast prior to blood draw. Non-HDL was calculated as the difference between total cholesterol and HDL, which may be a superior predictor of cardiovascular events compared to LDL-c [28].

White blood cell (WBC) count was determined by the Coulter Counter Model S-PLUS JR with Coulter histogram differential, an automated hematology analyzer. Homocysteine (Hcy) level was determined by reverse-phase high-performance liquid chromatography (HPLC) and fluorescence detection [29]. Due to NHANES limitations, Hcy testing was performed only on participants ≥12 years of age in phase II (1991–1994).

Fibrinogen was measured by the Coagamate XC plus automated coagulation analyzer (Organon Teknika, Durham, NC). Ferritin was measured using the Bio-Rad Laboratories “Quantimune Ferritin IRMA” 1000 kit (Hercules, CA). Hemoglobin A1c was measured by the Bio-Rad DIAMAT glycosylated hemoglobin analyzer system, using HPLC principles (Bio-Rad Laboratories, Hercules, CA).

Serum C-Reactive Protein (CRP) is a marker of systemic inflammation and a validated independent risk factor for CHD [30]. It was quantified via latex-enhanced nephelometry using the Behring Nephelometer Analyzer System (Behring Diagnostics Inc., Somerville, NJ). Since most participants had CRP values below the lowest detectable level (20.95 nmol/L), CRP was dichotomized into low (<95.24 nmol/L) and high (≥95.24 nmol/L) levels which are consistent with previous NHANES III categorization [31, 32]. Low levels were used to define the non-inflammatory COPD group and high levels were used to define the inflammatory COPD group.

Diagnostic groups used for analyses. Participants were divided into five exposure groups based upon their COPD status from spirometry testing, their CRP level, and their self-reported CHD status. Participants whose spirometry results indicated no COPD were placed into Group 1- no COPD and no CHD *(“Healthy”individuals free of hypertension, diabetes, and arthritis*); participants who had COPD and had no inflammation (CRP <95.24 nmol/L) [31, 32] were included in Group 2 *(“non-Inflammatory COPD”)*; participants who had COPD and had inflammation (CRP ≥95.24 nmol/L) [31, 32] were included in Group 3 *(“inflammatory COPD”)*; participants who reported “yes” to the question “Has a doctor ever told you that you had a heart attack?” and did not have COPD based on spirometry results were placed in Group 4 (the *“CHD”* Group); and participants who reported “yes” to the question “Has a doctor ever told you that you had a heart attack?” and had COPD based on spirometry results were placed in Group 5 (the *“CHD + COPD”* Group).

### Statistical analysis

Descriptive statistics were used to examine demographics, CHD risk factors and inflammatory biomarkers of the five study groups. Analysis of variance and chi-square tests were used to evaluate differences in prevalence of continuous and categorical risk factors, respectively, among the five study groups while adjusting for the complex sampling design in NHANES III.

Cox proportional hazards models were used to examine the time to CHD mortality. In addition, we explored the relation between COPD subgroups and CVD as well as COPD subgroups and all-cause mortality after adjusting for the confounding effect of CHD risk factors and inflammatory biomarkers including age, gender, race/ethnicity, family history of CHD, diabetes, BMI, physical activity level, cholesterol ratio, current smoking, household income, education, and blood pressure medications. Tests for violations in the proportional hazards assumptions were also conducted and found to be insignificant.

The adjusted hazards ratios and their 95% confidence intervals were used to measure the association between COPD subgroups and (CHD, CVD and all-cause) mortality. Statistical analyses were performed using SAS-callable (version 9.2, SAS Institute Inc., Cary, NC) SUDAAN version 11.0.1 (Research Triangle Institute, Research Triangle Park, NC).

## Results

We first examined the characteristics of the five study groups that are presented in Table 1. The CHD only group included predominantly males (62.4%) whereas the “inflammatory” COPD group included mostly females (54.6%). Age, race, education, insurance status, income, employment, and smoking status were significantly different among the five groups (group p < 0.001). Only marital status and alcohol were non-significant among the five groups. Regarding smoking, the Healthy group had the lowest prevalence of ever smokers (52.8%). For the remaining groups, the CHD + COPD group had the highest prevalence of ever smokers (88.7%) while the CHD group had the lowest prevalence of ever smokers (65.4%) (p < 0.001). The non-inflammatory and inflammatory COPD groups, however, had more current smokers than the CHD group (29.0%, 38.6% and 17.4%, respectively, p <0.001). In terms of respiratory symptoms (cough or phlegm 3+ months), individuals in the inflammatory COPD group self-reported more symptoms (29.1%) compared to the non-inflammatory COPD group (17.1%), p = 0.028, despite nearly equivalent FEV1/FVC ratios.

**Table 1:** **Characteristics of the five study groups**

		“Healthy”	Non-inflammatory COPD	Inflammatory COPD	CHD only	CHD + COPD	Group p-value
N		2,107	1,100	118	235	121	
Weighted N		26,684,256	11,704,624	1,147,035	1,992,045	1,109,152	
Gender (%)	Male	47.9	57.0	45.4	62.4	73.7	<0.001
	Female	52.1	43.0	54.6	37.6	26.3	
Age (%)	40-49	56.8	18.8	5.8	12.3	3.3	<0.001
	50-59	24.6	22.2	24.6	20.3	13.5	
	60-69	11.3	28.5	29.1	27.7	28.9	
	70-79	5.5	22.4	33.7	28.6	36.4	
	80+	1.8	8.2	6.8	11.1	18.0	
	60+ years	18.6	59.1	69.6	67.4	83.3	<0.001
Race (%)	White	85.1	91.0	83.7	86.0	96.7	<0.001
	Black	6.8	5.1	8.7	9.2	2.5	
	Hispanic	8.0	3.9	7.6	4.8	0.8	
	Minority	14.8	9.0	16.3	15.0	3.3	
Education (%)	< High School	18.9	30.4	36.0	36.0	40.7	<0.001
Marital Status (%)	Partnered	76.8	72.9	62.9	74.6	72.3	0.138
Insured (%)		92.4	95.6	98.9	97.1	97.5	<0.001
Income (%)	<$20 K/year	19.8	35.5	50.9	42.3	41.9	<0.001
Employed (%)		77.6	47.6	32.7	38.1	28.2	<0.001
Any Alcohol Consumption (%)		89.0	89.9	90.5	86.6	87.9	0.727
Alcohol * Consumption	(# drinks per week)	3.8 (0.3)	3.9 (0.5)	6.7 (3.3)	2.8 (0.7)	3.6 (1.3)	0.466
Smoking Status (%)	Never	47.2	27.2	23.3	34.6	11.3	<0.001
	Past	34.6	43.8	38.2	48.0	69.3	
	Current	18.2	29.0	38.6	17.4	19.4	
	Ever (Past + Current)	52.8	72.8	76.8	65.4	88.7	<0.001
No. cigarettes per day*		3.5 (0.3)	6.7 (0.5)	7.9 (1.5)	3.9 (1.2)	5.3 (1.9)	<0.001
10 pack-year smoking history (%)		30.4	55.1	61.2	45.4	71.9	<0.001
Exposure to ETS (%)		30.4	38.0	46.9	28.3	29.0	0.004
Cough or phlegm, 3+ months (%)		7.9	17.1	29.1	10.8	19.5	<0.001
Shortness of breath (%)		16.5	34.4	50.3	54.6	48.9	<0.001
FEV1/FVC ratio*		0.79 (0.01)	0.63 (0.01)	0.61 (0.01)	0.78 (0.01)	0.62 (0.01)	<0.001
FEV1 (ml)*		3196 (26.6)	2417 (39.2)	1952 (94.3)	2699 (82.3)	2176 (75.8)	<0.001
FVC (ml)*		4069 (33.8)	3823 (57.3)	3165 (128.7)	3494 (109.5)	3503 (95.9)	<0.001
COPD Severity (%)	None	100.0	0.0	0.0	100.0	0.0	<0.001
	Mild	0.0	58.7	40.5	0.0	46.1	
	Moderate	0.0	35.2	45.3	0.0	49.0	
	Severe	0.0	6.1	14.2	0.0	4.9	
Mortality (%)	CHD	2.4	6.4	12.7	30.1	33.5	<0.001
	CVD	3.5	9.8	15.7	33.5	39.7	<0.001
	All-cause	11.3	40.4	57.7	62.8	69.2	<0.001
No. months follow-up*		173 (2.8)	150 (3.6)	128 (7.3)	124 (5.6)	115 (6.9)	<0.001

*mean ± standard error.

CHD risk factors and inflammatory biomarkers among the five study groups are presented in Table 2. All variables were significantly different among the four groups. Among the five groups, body mass index (BMI) was lowest in the non-inflammatory COPD group while hypertension and diabetes prevalence rates and triglycerides and TC/HDL levels were significantly higher in the CHD + COPD group compared to the COPD groups, except for HDL. HDL, which has an inverse relation, was lower in the CHD + COPD group compared to the COPD groups. Of the inflammatory biomarkers, CRP, ferritin, fibrinogen, and WBC were elevated in the inflammatory COPD group (p < 0.001).

**Table 2:** **CHD risk factors and inflammatory biomarkers among the five study groups**

		“Healthy”	Non-inflammatory COPD	Inflammatory COPD	CHD Only	CHD + COPD	Group p-value
Age*		50.9 (0.5)	62.2 (0.7)	65.6 (1.3)	65.1 (1.1)	69.6 (1.0)	<0.001
BMI*		26.4 (0.1)	26.2 (0.2)	27.9 (0.7)	28.5 (0.8)	26.4 (0.3)	0.002
Physical Activity (METS/week)*		116 (4.4)	108 (5.8)	66 (8.5)	128 (12.8)	123 (17.7)	0.008
HTN (%)		0.0	27.2	43.6	54.4	55.3	<0.001
SBP		122 (0.4)	133 (0.8)	139 (2.1)	138 (1.4)	139 (2.5)	<0.001
DBP		75 (0.3)	76 (0.4)	75 (1.1)	75 (0.9)	73 (1.4)	0.373
Diabetes (%)		0.0	7.1	4.4	20.4	15.9	<0.001
HbA1c		5.28 (0.03)	5.62 (0.05)	5.83 (0.11)	5.98 (0.08)	5.90 (0.14)	<0.001
Framingham Risk (%)	<10%	82.2	56.5	52.4	38.5	29.4	<0.001
	10-20%	16.3	33.4	29.1	46.0	50.8	
	>20%	1.5	10.0	18.5	15.5	19.8	
Risk Group (%)	CHD Equivalent	1.5	18.2	22.1	100.0	100.0	<0.001
	High	11.9	19.4	18.5	0.0	0.0	
	Moderate	14.6	21.0	20.2	0.0	0.0	
	Low	71.0	40.7	36.4	0.0	0.0	
	High+CHD	13.4	37.6	40.6	100.0	100.0	<0.001
Lipid Profiles*							
Total cholesterol	(mmol/L)	5.50 (0.04)	5.66 (0.04)	5.53 (0.10)	5.85 (0.10)	5.85 (0.09)	<0.001
HDL	(mmol/L)	1.34 (0.02)	1.32 (0.02)	1.34 (0.07)	1.20 (0.05)	1.18 (0.04)	0.002
Triglycerides	(mmol/L)	1.57 (0.04)	1.71 (0.04)	1.86 (0.11)	2.07 (0.09)	2.26 (0.19)	<0.001
TC/HDL ratio		4.47 (0.08)	4.67 (0.09)	4.67 (0.22)	5.30 (0.20)	5.53 (0.31)	<0.001
Non-HDL	(mmol/L)	4.15 (0.04)	4.15 (0.04)	4.22 (0.12)	4.65 (0.11)	4.67 (0.09)	<0.001
Inflammatory Biomarkers*							
Homocysteine	(umol/L)	9.45 (0.23)	11.59 (0.58)	12.85 (1.86)	11.94 (0.55)	11.58 (0.65)	<0.001
CRP	(mmol/L)	30.14 (1.19)	27.87 (0.71)	200.27 (14.57)	62.98 (11.46)	73.92 (20.11)	<0.001
Ferritin	(pmol/L)	296.09 (11.16)	355.37 (12.83)	373.77 (37.54)	334.66 (29.30)	342.82 (43.24)	<0.001
Fibrinogen	(umol/L)	8.30 (0.10)	8.78 (0.11)	12.21 (0.50)	9.48 (0.27)	9.75 (0.42)	<0.001
WBC	(x10^3)	6.71 (0.06)	7.37 (0.14)	8.45 (0.33)	7.47 (0.24)	7.66 (0.32)	<0.001

*mean ± standard error.

CRP was significantly elevated in the inflammatory COPD group (200.27 nmol/L) compared to the CHD group (62.98 nmol/L) and CHD + COPD group (73.92 nmol/L) (p <0.001). Additionally, fibrinogen and WBC were also elevated in the inflammatory COPD group compared to the CHD and the CHD + COPD group (p <0.001 AND P = 0.009, respectively) and the non-inflammatory COPD group (p <0.001 and p = 0.005).

The hazard ratios (HRs) of CHD mortality, CVD and all-cause mortality among the five study groups are presented in Table 3. In the fully-adjusted models, the risk for CHD mortality were similar for the “Healthy” and COPD without inflammation groups, intermediate risk for the COPD with inflammation group (HR 2.06, 95% CI 0.84-5.04), and higher for the COPD + CHD group (HR 3.89, 95% CI 2.28-6.65) and CHD group (HR 4.55, 95% CI 2.48-8.36). A similar pattern was found for CVD mortality. For all-cause mortality, the risk was significant and similar for the COPD with inflammation group (HR 2.01, 95% CI 1.24-3.28), the CHD group (HR 2.48, 95% CI 1.94-3.16) and the COPD and CHD group (HR 2.19, 95% CI 1.63-2.95).

**Figure 1:**
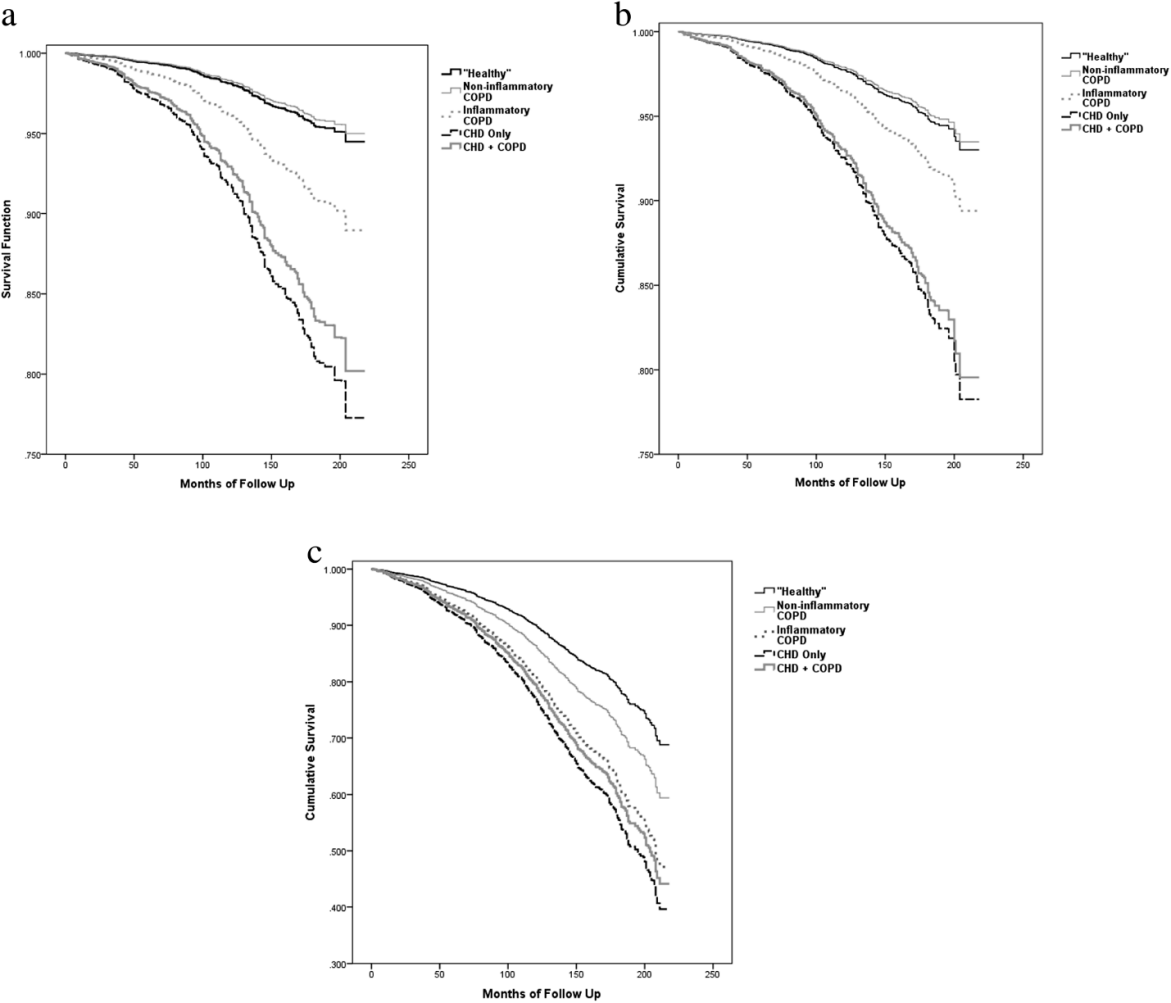
**Adjusted survival curves by mortality category in NHANES III by CHD/COPD diagnosis group. a**. CHD death. **b**. CVD death. **c**. All-cause mortality.

Figures 1a, b and c present the covariate adjusted Cox proportional hazards model survival curves for CHD, CVD, and all-cause mortality respectively among the five study groups. The mortality rate curves for both CHD and CVD were similar and were accelerated for the CHD and CHD + COPD groups followed by the inflammatory COPD group. For all-cause mortality, the mortality rate curves were accelerated for CHD, CHD + COPD, and COPD with inflammation compared to non-inflammatory COPD and the “Healthy” group

**Table 3:** **Hazards ratios and associated 95% CI for CHD, CVD, and all-cause mortality survival models**

Model	“Healthy” (n=2107) [L1]	Non-inflammatory COPD (n=1100) [L2]	Inflammatory COPD (n=118) [L3]	CHD only (n=235) [L4]	COPD + CHD (n=121) [L5]	Group comparison p-values		
						[L2] vs [L3]	[L3] vs [L4]	[L4] vs [L5]
CHD Mortality								
Crude	1.00	3.20 (2.22-4.61)	7.81 (3.10-19.66)	18.91 (11.85-30.19)	23.20 (12.85-41.89)	0.043	0.044	0.426
Age Adjusted	1.00	1.21 (0.81-1.82)	2.63 (0.96-7.20)	6.36 (3.72-10.87)	6.29 (3.58-11.03)	0.102	0.065	0.961
Non-modifiable Adjusted	1.00	1.11 (0.75-1.65)	2.52 (0.92-6.89)	5.56 (3.24-9.55)	5.02 (2.83-8.90)	0.082	0.098	0.671
Full Model	1.00	0.90 (0.61-1.33)	2.06 (0.84-5.04)	4.55 (2.48-8.36)	3.89 (2.28-6.65)	0.061	0.112	0.498
CVD Mortality								
Crude	1.00	3.37 (2.38-4.79)	6.60 (2.93-14.85)	14.53 (9.60-22.00)	18.81 (11.14-31.76)	0.080	0.029	0.269
Age Adjusted	1.00	1.21 (0.83-1.79)	2.11 (0.87-5.08)	4.62 (2.96-7.21)	4.78 (3.04-7.52)	0.182	0.051	0.861
Non-modifiable Adjusted	1.00	1.15 (0.78-1.68)	2.01 (0.84-4.79)	4.25 (2.70-6.69)	4.12 (2.60-6.54)	0.164	0.056	0.880
Full Model	1.00	0.93 (0.62-1.40)	1.55 (0.68-3.51)	3.38 (1.97-5.80)	3.16 (1.99-5.01)	0.202	0.065	0.727
All-Cause Mortality								
Crude	1.00	4.30 (3.51-5.26)	7.55 (4.65-12.25)	8.55 (6.61-11.07)	10.30 (7.21-14.71)	0.007	0.561	0.299
Age Adjusted	1.00	1.80 (1.44-2.25)	2.79 (1.63-4.79)	3.25 (2.59-4.08)	3.17 (2.33-4.30)	0.061	0.523	0.847
Non-modifiable Adjusted	1.00	1.70 (1.36-2.12)	2.72 (1.62-4.56)	2.96 (2.33-3.76)	2.72 (1.97-3.74)	0.037	0.701	0.546
Full Model	1.00	1.39 (1.12-1.74)	2.01 (1.24-3.28)	2.48 (1.94-3.16)	2.19 (1.63-2.95)	0.096	0.373	0.348

Non-modifiable adjusted model covariates: age, gender, race/ethnicity, family history CHD.

Full model covariates: age, gender, race/ethnicity, household income, education, family history CHD, diabetes, BMI, physical activity level, SBP, cholesterol ratio, current smoking, BP medications.

## Discussion

To our knowledge, this is the first study to examine the association between subgroups of COPD and CHD, CVD and all-cause mortality. Our findings suggest that CHD, CVD and all-cause mortality rates did not differ significantly between individuals with CHD and individuals with CHD + COPD. Although the mortality rates for individuals with COPD with inflammation were lower, of interest was that the inflammatory COPD group included mainly women who, despite having similar pulmonary function test parameters as the non-inflammatory COPD group, experienced more cough and phlegm production. Additionally, they were less physically active and had elevated inflammatory biomarkers compared to those with CHD and those with CHD + COPD. Furthermore, a consistent trend of monotonically increasing CHD and CVD mortality rates was observed when the “Healthy”, non-inflammatory COPD, inflammatory COPD, CHD + COPD and CHD groups were compared in that order.

The hazard ratios were not significantly different between the CHD group and the CHD + COPD group and were greater compared to individuals with COPD with inflammation. In contrast, all-cause mortality was equal among those in the inflammatory COPD group, the CHD group, and the CHD + COPD group. However, given the small number of individuals in the inflammatory COPD group (n = 118), we may not have had sufficient power to examine the association for CHD and CVD which requires further investigation.

Survival curve analysis also showed that inflammatory COPD, CHD only, and CHD + COPD follow virtually the same curve for all-cause mortality. Inflammatory COPD had a worse survival curve compared to non-inflammatory COPD in the all-cause mortality model, suggesting that those with inflammatory COPD have an accelerated mortality rate compared to individuals with non-inflammatory COPD.

In a previous study of NHANES III participants who were fifty years of age and older, Sin and Man examined whether CRP and other systemic inflammatory markers were present in individuals with chronic airflow obstruction that were also associated with cardiac injury (as measured by a Cardiac Infarction Injury Score based on EKG findings). They reported that severe airway obstruction, in the setting of elevated CRP, increased risk of cardiac injury almost two-fold [12]. Their findings suggest “that the systemic inflammation associated with moderate to severe airflow obstruction and an increased risk of cardiac injury may partially explain the high rates of CVD complication in COPD” [12].

Our study did not confirm this finding except for total mortality. However, our study differs in that we included participants ≥40 years old while Sin and Man included those ≥50 years old and used EKG criteria as a measure of heart disease rather than measuring mortality as the outcome. Furthermore, as previous mentioned, our sample included only a small number of individuals in the inflammatory COPD group and we may not have had sufficient power to examine this association.

### Inflammation a possible underlying factor

As mentioned earlier, COPD is increasingly being identified as a systemic disease with comorbidities extending beyond the lungs. These systemic sequelae include cardiovascular disease, diabetes, osteoporosis, anemia, cachexia, hypertension, cancer and depression [33]. Systemic inflammation may be the common pathway among these diseases, explaining their high rate of prevalence together [33]. Evidence for systemic inflammation is rapidly expanding. COPD patients exhibit elevated levels of inflammatory biomarkers including CRP, TNF-α, fibrinogen, leukocytes, IL-6, and IL-8 [34, 35] suggesting that COPD may be similar to other diseases associated with systemic inflammation including CAD, peripheral arterial disease, osteoporosis and diabetes [14]. In fact, the systemic inflammation may initiate or worsen the comorbid diseases and may potentiate the morbidity of COPD that could lead to an increase in total mortality which we observed in this study.

Current goals of COPD treatment include decreasing exacerbation frequency, improving symptoms and raising exercise tolerance [25]. Medications used to achieve these ends include inhaled β-2 agonists, inhaled anticholinergics, corticosteroids, theophylline, and oxygen [25]. This paradigm, however, ignores potentially large gains in mortality and comorbidity reduction by focusing treatment solely on the lung. Given the increased risk associated with COPD, therapeutic interventions may help to improve cardiovascular mortality.

If systemic inflammation is a common pathway for both CHD and COPD with inflammation, one approach may be expanded use of statins. Besides lowering cholesterol, statins display anti-inflammatory and immunomodulatory effects [36]. The benefits of statin therapy in individuals with normal lipid values but systemic inflammation have been recently demonstrated in the JUPITER Study. They found that rosuvastatin significantly reduced the incidence of cardiovascular events (HR = 0.56, 95% CI 0.46-0.69, p <0.0001), alluding to the potential anti-inflammatory benefit of statins [37].

Whether this benefit may also be observed in COPD patients was studied by Mancini et al. who showed a significant reduction in mortality among high-risk COPD patients on statins (RR 0.50, 95% CI 0.40-0.62; p <0.0001) and an improved benefit among those taking both statins and an ACEI/ARB (RR 0.42, 95% CI 0.33-0.52, p < 0.0001) [38]. These benefits persisted among COPD patients regardless of steroid use [38]. This finding was corroborated by Mortensen et al. who showed a decrease in 90-day mortality post-discharge after COPD exacerbation in patients using statins (OR 0.51, 95% CI 0.40-0.64) and ACEI/ARB (OR 0.55, 95% CI 0.46-0.66) prior to admission [39].

### Study strengths and limitations

Our study was limited by several factors. Mortality data was obtained through the National Death Index and the cause of death reported on death certificates may suffer from inaccuracies or misclassification. Current GOLD guideline recommendations for spirometry-based diagnosis of COPD have been criticized for not adequately accounting for age-related changes in lung function and inter-subject spirometry performance variability, resulting in misdiagnosis in the elderly [40]. By using GOLD guidelines, we were subject to this possibility.

Additionally, since the diagnosis of COPD was based on pre-bronchodilator spirometry, it is possible that there may have been an overestimation of COPD. However, this overestimation was probably distributed equally across the five diagnostic groups. We were limited by the NHANES data set and used mortality as a measure for CHD risk instead of new CHD events (i.e. MI), which may be of future interest. Finally, as previously mentioned, we note a small sample size (n = 118) in the inflammatory COPD subgroup. The sample, however, represents a weighted n = 1,147,035 people in the US. Future work should investigate whether this finding is replicable in other cohorts.

Given that NHANES III is a population-based survey, one strength of our study is that our results are representative of the US population and are thereby generalizable. NHANES III also oversampled African Americans and Hispanics to allow better estimation of the true US population. Another strength is the inclusion of numerous CHD risk factors and inflammatory biomarkers in our analysis.

## Conclusions

Our findings did not show that inflammatory COPD may be a CHD risk equivalent but due to the small sample requires additional investigation. Additionally, given that COPD results from inflammation, the systemic inflammation associated with COPD may have worsened comorbid conditions and may have lead to the increased total mortality found in the COPD with inflammation and CHD + COPD groups which requires further investigation. Furthermore treatment of COPD may require not only treatment of the systematic inflammation but also treatment of the associated comorbidites.
